# Traumatic atlantoaxial anteroinferior subluxation with dens and Hangman fractures: A case report

**DOI:** 10.1097/MD.0000000000024396

**Published:** 2021-01-22

**Authors:** Sung-Kyu Kim, Dong-Gune Chang, Jong-Beom Park, Hyoung-Yeon Seo, Yuna Kim

**Affiliations:** a Department of Orthopaedic Surgery, Chonnam National University Medical School and Hospital, Gwangju; b Department of Orthopaedic Surgery, Sanggye Paik Hospital, College of Medicine, Inje University; c Department of Orthopaedic Surgery, College of Medicine, the Catholic University of Korea, Seoul, Korea.

**Keywords:** atlantoaxial anteroinferior subluxation, dens fracture, Hangman fracture

## Abstract

**Rationale:**

Traumatic atlantoaxial anteroinferior subluxation associated with a dens fracture and a Hangman fracture is a very rare and complex injury. Therefore, appropriate surgical strategy is not established.

**Patient concerns:**

An 85-year-old female presented with posterior neck pain and atypical neck position caused after rolling down a hill. Although neurological examinations for motor, sensory, gait, and reflex tests were normal, the patient complained of an abnormal neck posture.

**Diagnoses:**

Radiological examinations revealed an atlantoaxial anteroinferior subluxation with kyphosis, a type IIA dens fracture (Anderson and D’Alonzo classification) with an anterolateral rotatory angulation of type IIA dens fracture fragment, and a type I Hangman fracture (Levine and Edwards classification). Nevertheless, the transverse atlantal ligament was intact.

**Interventions:**

We considered that the intact transverse atlantal ligament and kinking of the type IIA dens fracture fragment into the left lateral mass of C1 prevented a spinal cord injury by blocking a further displacement of C1 to C2. Due to the patient's osteoporosis and the anterolateral rotatory angulated type IIA dens fracture fragment, a forceful reduction of the atlantoaxial anteroinferior subluxation with kyphosis could pose a high risk of fixation failure and spinal cord injury. Therefore, we performed in-situ posterior C1-2 fusion using a C1 lateral mass screw and C2 lamina screw fixations.

**Outcomes:**

At 1 year after surgery, the bone union of all fractures was achieved in the kyphosis state. Furthermore, the patient's clinical symptoms were improved with no neurological deficit.

**Lessons:**

A thorough radiological examination and appropriate surgical strategy are important for successful diagnosis and treatment of a complex C1-2 injury.

## 1 Introduction

Typically, a C2 fracture occurs as a dens fracture or a Hangman fracture,^[1,2]^ either of which occurs alone or in combination with other spinal injuries.^[3]^ Reportedly, the combination of dens fracture, Hangman fracture, and other fracture/dislocation/subluxation of C1 to C2 is a very rare and complex injury.^[4]^ Thus, an appropriate treatment strategy is crucial to managing such a complex C1-2 injury successfully.

This report describes the radiological findings, treatment process, and results of the first case of atlantoaxial anteroinferior subluxation associated with a type IIA dens fracture (according to the Anderson and D’Alonzo classification), an anterolateral rotatory angulation of the type IIA dens fracture fragment, and a type I hangman fracture (according to the Levine and Edwards classification).

## 2 Case report

**Figure 1:**
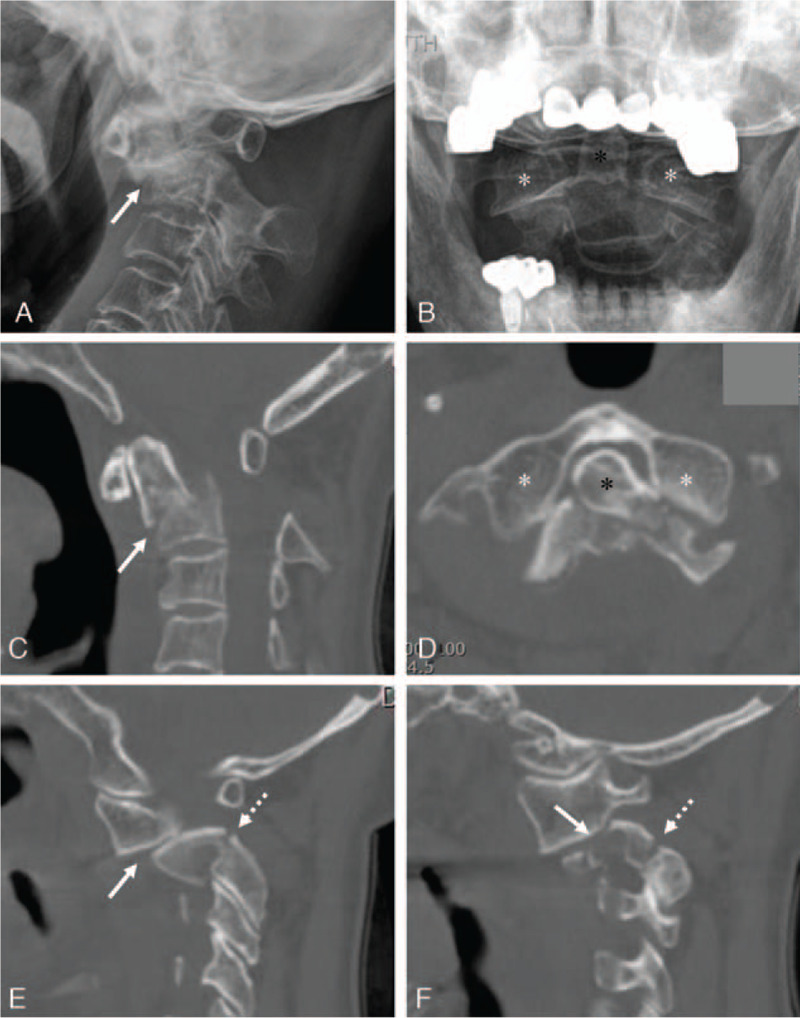
A lateral radiograph (a) and midline sagittal computed tomography (CT) (c) scan revealed the anteroinferior displacement of a type IIA dens fracture with kyphosis (white arrow). An open-mouth view (b) and axial CT scan (d) revealed an atlantoaxial anteroinferior subluxation (white asterisks) and anterolateral rotatory angulation of the type IIA dens fracture fragment (dark asterisk). The right (e) and left (f) parasagittal CT scans revealed fractures of both pars interarticularis (type I Hangman fracture) (dotted white arrows) and the left transverse foramen (white arrow).

**Figure 2:**
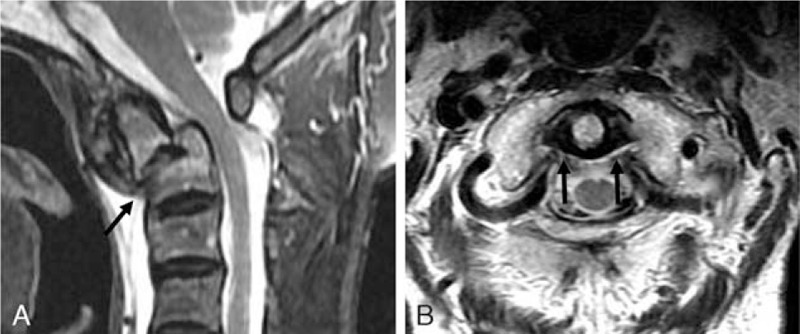
Sagittal magnetic resonance imaging (a) revealed the anteroinferior displacement of the type IIA dens fracture (dark arrow) (according to the Anderson and D’Alonzo classification) without spinal cord compression. Axial magnetic resonance imaging (b) revealed an intact transverse atlantal ligament (dark arrows).

An 85-year-old female visited our hospital with posterior neck pain that occurred after rolling down a hill 2 weeks ago. Although neurological examinations for motor, sensory, gait, and reflex tests were normal, the patient complained of an abnormal neck posture. A lateral radiograph (Fig. 1a) and midline sagittal computed tomography (CT) (Fig. 1c) scan revealed the anteroinferior displacement of a type IIA dens fracture with kyphosis. An open-mouth view (Fig. 1b) and axial CT scan (Fig. 1d) revealed an atlantoaxial anteroinferior subluxation and anterolateral rotatory angulation of the type IIA dens fracture fragment. The right (Fig. 1e) and left (Fig. 1f) parasagittal CT scans revealed fractures of both pars interarticularis and the left transverse foramen. Sagittal magnetic resonance imaging (Fig. 2a) revealed the anteroinferior displacement of the type IIA dens fracture without spinal cord compression. Axial magnetic resonance imaging ( (Fig. 2b) revealed an intact transverse atlantal ligament (TAL).

**Figure 3:**
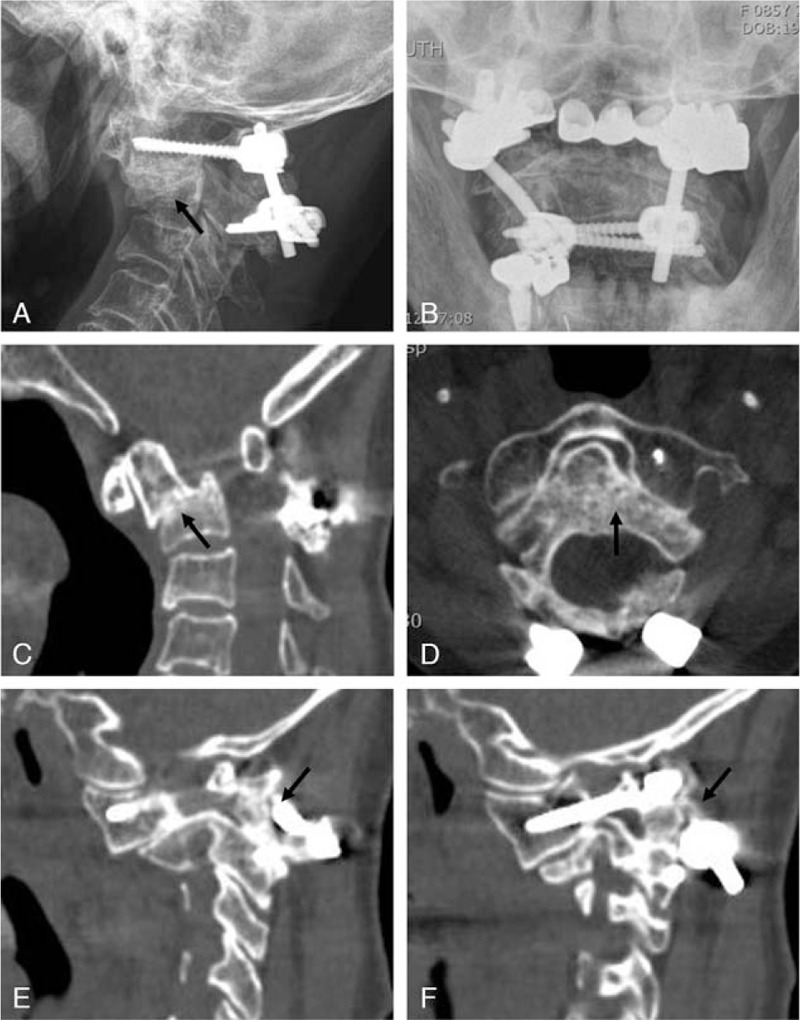
One year after posterior C1-2 fusion surgery, lateral radiograph (a) and midline sagittal computed tomography (CT) (c) show the union (dark arrows) of the type IIA dens fracture in a kyphotic state. An open-mouth view (b) and axial CT scan (d) reveals the union of the type IIA dens fracture fragment at an anterolateral rotatory angulated state (dark arrow). The right (e) and left (f) parasagittal CT scans demonstrate the fracture union of both pars interarticularis (type I Hangman fracture) and left transverse foramen (dark arrows).

Due to the patient's old age, the bone quality of the patient was osteoporotic. The atlantoaxial anteroinferior subluxation remained overlooked for 2 weeks after injury. Besides, the anterolateral rotatory angulated type IIA dens fracture fragment was kinked into the dens fracture fragment into the left lateral mass of C1. We considered that a forceful reduction of the atlantoaxial anteroinferior subluxation with kyphosis to a neutral or lordotic state could cause a high risk of fixation failure and spinal cord injury. Therefore, we performed in-situ posterior C1-2 fusion with autogenous iliac crest bone graft. Notably, C1 lateral mass screws were successfully inserted for C1 fixation. We used C2 lamina screws for C2 fixation rather than C2 pedicle screws because of fractures of both pars interarticularis and type IIA dens fracture. Postoperatively, the patient wore the Philadelphia brace for 3 months. After 1 year of surgery, follow-up plain radiographs and CT scans (Fig. 3) revealed the bone union of type IIA dens fracture and fractures of both pars interarticularis in a kyphotic state with good maintenance of the C1-2 rods and screws. Moreover, the patient's clinical symptoms were improved with no neurological deficit.

## 3 Discussion

This study reported a first case of atlantoaxial anteroinferior subluxation associated with a dens fracture and a Hangman fracture. Compared with previously reported cases, this case is unique for several reasons. Firstly, to the best of our knowledge, an anterolateral rotatory angulation of the type IIA dens fracture fragment has not been reported before. Secondly, despite the atlantoaxial anteroinferior subluxation with kyphosis and multiple C2 fractures, the patient had no neurological deficit. We believe that the intact TAL and kinking of the type IIA dens fracture fragment into the left lateral mass of C1 prevented a further displacement of C1 to C2 and, thus, avoided spinal cord injury. If the TAL had not been intact, a sagittal posterior angulation of the type IIA dens fracture fragment would have occurred; in this scenario, chances of cord compression of the patient would have been extremely high. Undeniably, the wide spinal canal in C1 to C2 was also one of the reasons for the absence of neurological deficit.

The appropriate treatment strategy of traumatic atlantoaxial anteroinferior subluxation or dislocation with multiple C2 fractures has not been established because this combined C1-2 injury is a very rare and complex. According to previous studies, trial of reduction of atlantoaxial anteroinferior subluxation or dislocation by skull traction is recommended. If closed reduction fails, open reduction, and fusion should be performed. After successful closed reduction, if C1-2 is stable, conservative treatment, such as Halovest or rigid brace, is sufficient. However, if significant residual C1-2 instability is identified, posterior C1-2 fusion must be performed. The appropriate treatment strategy for atlantoaxial anteroinferior subluxation associated with multiple C2 fractures should be determined by considering several factors, such as C1-2 stability and stability of each associated C2 fracture, comprehensively.

A type IIA dens fracture is a variant type II dens fracture caused by an additional comminuted fracture fragment at the base of dens^[5–7]^; as it is considered an unstable fracture, surgical treatment is recommended. A type I Hangman fracture is a stable fracture and well treated with conservative treatment.^[8,9]^ However, our patient presented with a complex C1-2 injury because the combination of a type IIA dens fracture and a type I Hangman fracture is accompanied by an atlantoaxial anteroinferior subluxation and the anterolateral rotatory angulation of the type IIA dens fracture fragment. Thus, the definite treatment strategy of our case has not been established yet. In cases of dens fracture with kyphosis, surgery is usually performed after the reduction of kyphosis by skull traction or halo vest. However, prolonged skull traction or Halo vest fixation to achieve the reduction of kyphosis could lead to several complications in elderly patients.^[5,6]^

Due to the patient's osteoporosis and anterolateral rotatory angulated type IIA dens fracture fragment, we assumed that a forceful reduction of the atlantoaxial anteroinferior subluxation with kyphosis to a neutral or lordotic state could cause a high risk of fixation failure and spinal cord injury.^[10–12]^ Notably, C2 pedicle screw fixation is biomechanically stronger than C2 lamina screw fixation.^[4]^ However, we deemed the insertion of C2 pedicle screws technically challenging because of fractures of both pars interarticularis and the type IIA dens fracture. Thus, we performed in-situ posterior C1-2 fusion using C1 lateral mass and C2 lamina screw fixations without forceful reduction of the atlantoaxial anteroinferior subluxation with kyphosis. Finally, we achieved a successful union of all fractures, and the patient's clinical symptoms were improved without neurological deficit.

In conclusion, this study reports a first case of traumatic atlantoaxial anteroinferior subluxation associated with a type IIA dens fracture, an anterolateral rotatory angulation of the type IIA dens fracture fragment, and a type I Hangman fracture. A thorough radiological examination and appropriate surgical strategy are important for successful diagnosis and treatment of a complex C1-2 injury.

## Author contributions

**Conceptualization:** Jong-Beom Park.

**Data curation:** Hyoung-Yeon Seo.

**Investigation:** Sung-Kyu Kim, Hyoung-Yeon Seo, Yuna Kim.

**Methodology:** Dong-Gune Chang, Yuna Kim.

**Project administration:** Jong-Beom Park.

**Resources:** Jong-Beom Park, Yuna Kim.

**Supervision:** Jong-Beom Park.

**Validation:** Dong-Gune Chang, Jong-Beom Park, Hyoung-Yeon Seo.

**Visualization:** Dong-Gune Chang, Hyoung-Yeon Seo.

**Writing – original draft:** Sung-Kyu Kim.

**Writing – review & editing:** Dong-Gune Chang.
